# Transcriptional Response of Peripheral Blood Mononuclear Cells from Cattle Infected with *Mycobacterium bovis*

**DOI:** 10.1371/journal.pone.0041066

**Published:** 2012-07-16

**Authors:** Federico Carlos Blanco, Marcelo Soria, María Verónica Bianco, Fabiana Bigi

**Affiliations:** 1 Instituto de Biotecnología-CICVyA, Instituto Nacional de Tecnología Agropecuaria, Hurlingham, Buenos Aires, Argentina; 2 Agricultural Microbiology, School of Agronomy, Buenos Aires University, INBA-Consejo Nacional de Investigaciones Científicas y Técnicas, Ciudad de Buenos Aires, Argentina; Fundació Institut d’Investigació en Ciències de la Salut Germans Trias i Pujol. Universitat Autònoma de Barcelona. Ciberes, Spain

## Abstract

*Mycobacterium bovis* is the causative agent of most cases of bovine tuberculosis. The identification of bTB biomarkers in specific stages of the disease will contribute to a better understanding of the immunopathology associated with tuberculosis and will enable their use in disease diagnosis and prognosis. The aim of this study was to evaluate the gene expression profile induced after specific stimulation of bovine peripheral blood mononuclear cells from cattle infected with *M. bovis* using the Affymetrix® GeneChip® Bovine Genome Array. A total of 172 genes showed differential expression profile that was statistically significant with log2-fold change >2.5 and <−2.5. Twenty-four out of these genes were upregulated and 148 were downregulated in bovine peripheral blood mononuclear cells of *M. bovis*-infected cattle. The highest differentially-expressed genes were related to immune and inflammatory responses, apoptosis, endocytosis, cellular trafficking and genes encoding proteins involved in cellular matrix degradation. Microarray results were confirmed in another group of infected cattle by RT-qPCR for the *CD14*, *IL-1R*, *THBS1*, *MMP9* and *FYVE* genes. This study confirms previous findings that have shown that *M. bovis* infection in cattle results in the downregulation of immune response-related genes. Moreover, it validates the use of microarray platforms in combination with RT-qPCR to identify biomarkers of bovine tuberculosis. In addition, we propose CD14, IL-1R, THBS1, MMP9 and FYVE as potential biomarkers of bovine tuberculosis.

## Introduction

Bovine tuberculosis (bTB) is a serious animal and zoonotic disease that not only causes significant financial loss but is also a public health hazard. Although the main hosts of *Mycobacterium bovis*, the causative agent of bTB, are cattle, other animals, including humans, may be affected as well [1]. Infections in humans are associated with unpasteurized dairy product consumption and through contact to infected cattle. bTB is a factor that undermines the development of the dairy and meat industry and international commerce. *M. bovis* is closely related to *Mycobacterium tuberculosis*, the causative agent of human tuberculosis (TB), and both species are included in the *Mycobacterium tuberculosis* complex. In humans and cattle the disease is principally an infection of the respiratory system [2], [3], [4].

A large-scale transcriptional gene expression analysis has been used to define the repertoire of genes expressed in host-pathogen interactions between tuberculosis and many other infectious diseases [5]. This kind of transcriptional approach has significantly contributed to a better understanding of the mechanisms involved in these interactions, the cross-talk between intracellular pathogens and their host cells, and to identify novel mechanisms of bacterial evasion or immunological elimination. In addition, the characterization of the transcriptional profile associated with the resolution of infection or the development of disease may not only contribute to a deeper knowledge of the immunological parameters related to pathology but also to the identification of biomarkers that allow the prediction of disease outcome in cattle. The use of these biomarkers would be extremely useful for the detection of potential TB-transmitting animals in infected herds, which in turn may contribute to the control of bTB transmission between cattle and humans. Moreover, it has been proposed that the bovine is a robust animal model for preclinical safety and efficient evaluation of TB candidate vaccines targeting human population [6]. Therefore, biomarkers can also be used to anticipate the outcome of vaccine TB-protection assays in bovine models of infection. In this study, we aimed to evaluate the gene expression profile of bovine peripheral blood mononuclear cells (PBMCs) from cattle infected with *M. bovis* upon specific antigen stimulation. We found that more than 5,930 genes changed their level of expression upon infection of cattle with *M. bovis*. Further evaluation of RNA expression levels in PBMCs from cattle experimentally infected with *M. bovis* confirmed the microarray results for a subset of genes.

## Results

### DNA Microarray Analysis

Comprehensive gene expression profiles of six PBMC samples from *M. bovis*-infected calves and five PBMC samples from five healthy calves were generated with high-density oligonucleotide bovine arrays (Affymetrix) with 24,072 probe sets, which in total interrogated the expression levels of approximately 23,000 transcripts, some of which map to known genes.

A total of 5,920 probe sets passed the filtering step described in Materials and Methods (Figure S1). Limma’s linear models and empirical Bayes corrections for multiple comparisons were applied to assess the statistical significance of the expression profiles of the probe sets obtained in the previous step. Considering a threshold of 0.05 for the adjusted P value and an absolute value of log-2fold change equal to or greater than 2.5 (absolute value), 172 unique probe sets showed a differential expression that was statistically significant. Twenty-four out of these 172 unique probes were upregulated and 148 were downregulated in the PBMCs of *M. bovis*-infected cattle. A complete list of the differentially-expressed genes and the relative fold-change in expression between healthy and *M. bovis*-infected groups is provided in Table S1. Table 1 and 2 summarize these results. Data show that the highest differentially-expressed genes were mainly downregulated in PBMCs of *M. bovis*-infected cattle. Further inspection of these genes revealed that many of them are related to immune and inflammatory responses, apoptosis, endocytosis and cellular trafficking. In addition, some genes encoding proteins involved in cellular matrix degradation displayed downregulated expression in the PBMCs from the *M. bovis*-infected group (Table 1).

**Table 1:** The most relevant downregulated genes in PBMCs of *M. bovis* infected cattle.

Process	Symbol	FC	adj.P.Val	Gene name
**Immune response**				
	MRC1	−3.303	2.564E-13	Mannose receptor. C type 1
	CXCR2	−6.235	1.888E-11	Interleukin 8 receptor. Beta
	RETN	−6.201	9.116E-19	Resistin
	CSF1R	−4.440	5.873E-16	Colony stimulating factor 1 receptor
	CCL8	−4.272	6.349E-10	Chemokine (C-C motif) ligand 8
	CD14	−4.140	1.562E-17	CD14 molecule.
	FCAR	−4.129	6.639E-15	Fc fragment of IgA. receptor
	LOC508666	−3.963	1.659E-11	C-C motif chemokine 23-like
	CSF3R	−3.567	1.974E-09	Colony stimulating factor 3 receptor
	FCGR1A	−3.446	2.094E-15	Fc fragment of IgG. high affinity Ia. receptor (CD64)
	NRP1	−3.230	8.997E-10	Neuropilin 1
	IL1RN	−3.172	2.745E-18	Interleukin 1 receptor antagonist
	CCL2	−3.050	4.864E-18	Chemokine (C-C motif) ligand 2
	NFAM1	−3.046	7.780E-13	NFAT activating protein with ITAM motif 1/
	PF4	−3.038	9.815E-17	Platelet factor 4 (CXC chemokine family)
	IL13RA1	−2.963	1.893E-16	Interleukin 13 receptor. alpha 1
	CCR1	−2.936	4.864E-17	Chemokine (C-C motif) receptor 1
**Inflamation and apoptosis**				
	TGM3	−6.978		Transglutaminase 3
	TGFBI	−4.198	4.277E-10	TGF-β: Transforming growth factor beta
	TREM1	−4.011	3.437E-17	Triggering receptor expressed on myeloid cells
	PYCARD	−3.876	5.531E-16	PYD and CARD domain
	IL1R1	−3.866	8.108E-17	PYD and CARD domain
	HAS2	−3.663	2.858E-10	Hyaluronan synthase 2
**Metallopeptidases**				
	MMP9	−5.390	5.689E-18	Matrix metallopeptidase 9
	ECM1	−4.307	1.548E-16	Extracellular matrix protein 1
	MMP3	−4.302	4.349E-15	Matrix metallopeptidase 3
	ADAM8	−3.811	5.152E-17	ADAM metallopeptidase domain 8
	MMP19	−3.599	1.075E-13	Matrix metallopeptidase 19
	ADAMTSL4	−3.027	1.701E-16	Metalloproteinase with thrombospondin motifs
**Endocytosis and cellular traffiking**				
	SASH1	−4.934	1.167E-15	SAM and SH3 domain
	SH3BP4	−3.239	1.338E-13	SH3-domain binding protein 4
	VLDLR	−4.210	2.367E-17	Very low density lipoprotein receptor
	LOC533894	−4.154	3.107E-16	Low density lipoprotein receptor-related protein 1
	S100A9	−3.967	1.992E-17	S100 calcium binding protein A9
	ARHGAP21	−3.467	4.185E-16	Rho GTPase activating protein 21
**Others**				
	AREGB	−5.237	4.723E-13	Amphiregulin B (epidermal growth factor family)
	PLAUR	−3.134	7.737E-17	Plasminogen activator. urokinase receptor

FC: log2-fold change.

**Table 2:** The most relevant upregulated genes in PBMCs of *M. bovis* infected cattle.

Process	Symbol	FC	adj.P.Val	Gene name
**Immune response**	IFNG	4.972	3.600E-13	Interferon gamma
	BLA-DQB	4.049	3.238E-11	MHC class II antigen
	IL2	3.347	9.539E-14	Interleukin 2
	IL3	2.994	9.771E-13	Interleukin 3
	BOLA-DQA1	2.961	3.526E-09	Major histocompatibility complex class II.
	NOLC1	4.030	4.094E-17	Nucleolar and coiled-body phosphoprotein 1
**Other**	PSPH	2.974	3.257E-13	Phosphoserine phosphatase

FC: log2-fold change.

In order to better understand the biological significance of the differentially expressed genes, we compiled a second list of genes participating in pathways with differential expression by applying a Gene Set Enrichment analysis (GSEA) with the Bioconductor package GSEABase [7] (Table S2). Cellular pathways associated with the oxidative phosphorylation and T cell receptor signaling were among the most relevant pathways identified as upregulated in *M. bovis*-infected PBMCs (Table 3). This functional categorization analysis also revealed an enrichment of downregulated pathways involved in MAPK signaling, endocytosis, phagosome and lysosome functions, and regulation of actin cytoskeleton in the *M. bovis*-infected group (Table 3).

**Table 3:** Differential pathways between PBMC from *M. bovis*-infected animals and control animals using GSEA.

Downregulated pathways		
**KEGG**	**Path**	**url KEGG**
04010	MAPK signaling pathway	http://www.genome.jp/dbget-bin/www_bget?pathway:bta04010
04142	Lysosome	http://www.genome.jp/dbget-bin/www_bget?pathway:bta04142
04144	Endocytosis	http://www.genome.jp/dbget-bin/www_bget?pathway:bta04144
04145	Phagosome	http://www.genome.jp/dbget-bin/www_bget?pathway:bta04145
04722	Neurotrophin signaling pathway	http://www.genome.jp/dbget-bin/www_bget?pathway:bta04722
04810	Regulation of actin cytoskeleton	http://www.genome.jp/dbget-bin/www_bget?pathway:bta04810
05140	Leishmaniasis	http://www.genome.jp/dbget-bin/www_bget?pathway:bta05140
**Upregulated pathways**		
**KEGG**	**Path**	**url KEGG**
00190	Oxidative phosphorylation	http://www.genome.jp/dbget-bin/www_bget?pathway:bta00190
00230	Purine metabolism	http://www.genome.jp/dbget-bin/www_bget?pathway:bta00230
01100	Metabolic pathways	http://www.genome.jp/dbget-bin/www_bget?pathway:bta01100
03040	Spliceosome	http://www.genome.jp/dbget-bin/www_bget?pathway:bta03040
04660	T cell receptor signaling pathway	http://www.genome.jp/dbget-bin/www_bget?pathway:bta04660
05012	Parkinson’s disease	http://www.genome.jp/dbget-bin/www_bget?pathway:bta05012
05016	Huntington’s disease	http://www.genome.jp/dbget-bin/www_bget?pathway:bta05016
05200	Pathways in cancer	http://www.genome.jp/dbget-bin/www_bget?pathway:bta05200

The differential pathways were assessed with the gseattperm function in the Category Bioconductor package (10,000 permutations). All the pathways shown were significant at a P<0.01, adjusted for multiple comparisons using the false discovery rate method.

### Validation of Microarray Analysis by Real Time Quantitative PCR

**Figure 1:**
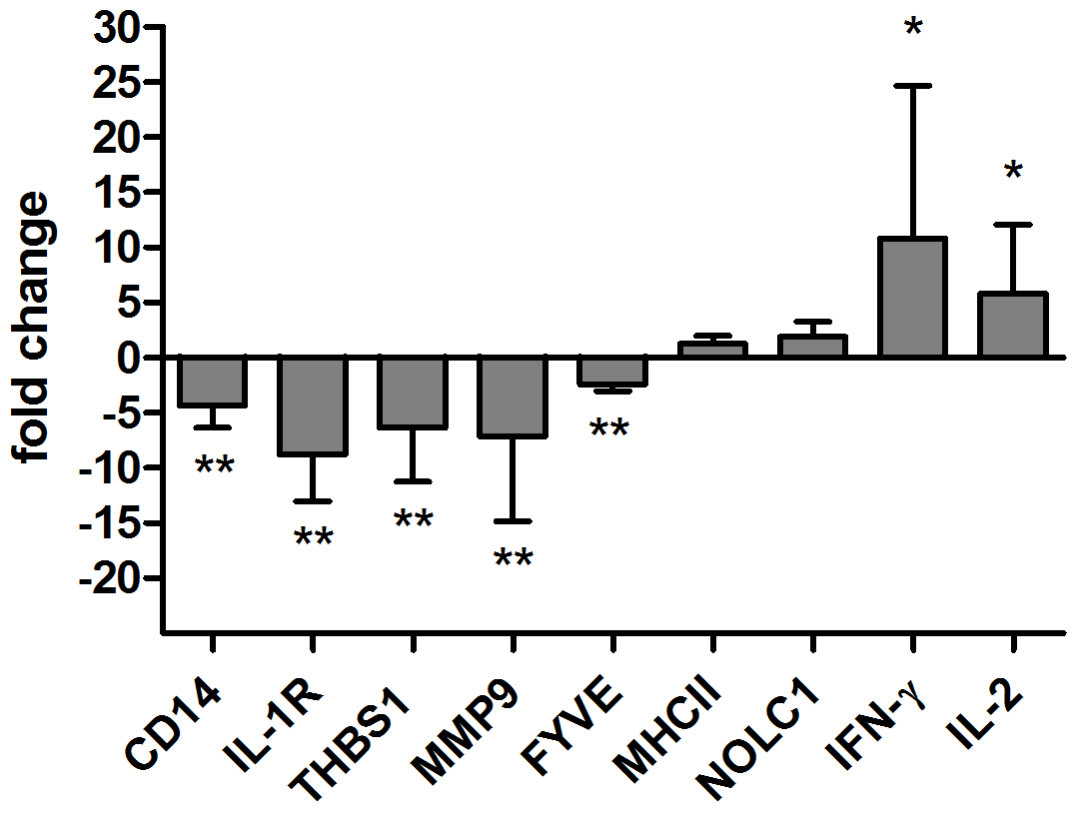
Gene expression fold-change differences between PBMC from *M. bovis*-infected animals (n = 8) and control animals (n = 8) using RT-qPCR. Relative gene expression was calculated using the 2-ΔΔCt method with E correction, using *gadph* mRNA expression as reference gene and the preimmune condition as the calibrator. Data were analyzed using a two-tailed unpaired t test (*p<0.05, **p<0.005). The bars indicate the average ratios of infected animals/uninfected animals ± SEM.

The microarray data for the genes whose expression varied at least two-fold between conditions were validated for a subset of six genes by RT-qPCR. One member of each of the most relevant functional categories was chosen for validation (Figure 1). To corroborate that the data set generated in the microarray analysis was not due to a bias in the sampled animals, the RT-qPCRs were performed in another set of experimentally infected cattle. PBMCs were collected from eight calves at 0 and 120 days post-experimental infection with *M. bovis*. With the exception of gene *CSF2* the RT-qPCR results validated the microarray data for the genes tested. As shown in Figure 1, the fold changes assessed by RT-qPCR were often greater than those determined by microarray analyses for the same genes (Tables 1 and 2, Table S1). As expected, the genes encoding IFNγ and IL2 cytokines, two biomarkers of infection, were significantly upregulated in the infected group. Although *NOLC1* and *MHCII* genes were also upregulated in the infected group, the differences were not statistically significant compared to the healthy group (only for one animal tested, these two genes were not upregulated). Thus, these results, together with those of the microarray experiment, indicate that the expression of *CD14, IL-1R, THBS1, MMP9* and *FYVE* is downregulated in cattle infected with *M. bovis*. In addition, the concordance between the microarray results and the RT-qPCR results supports the statistical approach adopted in this study.

In sum, these results show that the group of genes whose expression significantly changed in both the microarray and RT-qPCR analyses encodes proteins involved in inflammatory responses and intracellular trafficking.

## Discussion

In the current study, we compared the gene expression profile induced in PBMCs from *M. bovis*-infected cattle with that of healthy cattle. PBMCs represent an accessible tissue for the development of improved diagnostics. Moreover, previous studies have shown that the immune responses generated in the peripheral blood of cattle with tuberculosis reflect those elicited at the site of the disease [8]. This comparative transcriptional profile identified gene expression pathways involved in immune and inflammatory responses, apoptosis, endocytosis and cellular trafficking as highly downregulated in *M. bovis*-infected animals. Remarkably, the expression of several matrix metallopeptidases was significantly downregulated in infected animals. Only a few genes were significantly upregulated in infected animals and most of them encoded immune response-related proteins, such as IFNγ, interleukines, etc. Consistent with the findings of this study, it has been previously reported, in several studies, that the immuno-specific gene expression undergoes shutoff in *M. bovis*-infected cattle. Meade and coworkers have shown that the expression of several genes involved in the innate immune response was suppressed in *M. bovis*-infected cattle [9]. In addition, two consecutive studies of the same research group have reported that the balance in the transcriptional changes induced by *M. bovis* in different cell blood populations is suppression of gene expression [10], [9].

To determine the main biological processes associated with the differentially expressed genes, we clustered these genes in cellular pathways. In this analysis, we included all differentially-expressed genes that passed the statistical test without considering the fold changes. We observed statistically significant enrichment of induced genes in several pathways, but except for those encoding proteins involved in the T cell receptor signaling pathway, most of them were related to unspecific biological functions. In contrast, the repressed genes were mainly categorized in cellular trafficking pathways, such as endocytosis, phagosome, lysosome, MAP kinase signaling and regulation of the cytoskeleton, all functions associated with tuberculosis infections.

As mentioned above, the expression of four metallopeptidases was dowregulated in PBMCs of infected animals. Metalloproteinases (MMPs) were first described as enzymes involved in the degradation of the extracellular matrix. However, further studies have demonstrated that MMPs can modify many non-matrix substrates, such as cytokines and chemokines [11]. Chemokines play a central role in leukocyte recruitment to the site of infection, influencing the result of the inflammatory response. Proteolysis of chemokines by MMPs can either reduce or potentiate their activities. It has been reported that MMP3 inactivates several chemokines, while MMP9 increases the potency of at least two chemokines and inactivates several others [11]. The role of MMP9 in tuberculosis infection has been extensively studied [12], [13], [14], [15]. MMP9 is induced in cultured *M. tuberculosis*-infected monocytes [14] and in epithelial cells, and it promotes granuloma formation [15], [16].

*CD14* and IL-*1R* were among the inflammatory-response-related genes whose expression was highly repressed in PBMCs from *M. bovis*-infected cattle. It has been reported that CD14 mediates the *M. tuberculosis* TDM-induced proinflammatory response via SR/toll-like receptors 2 [17]. *IL-1R* encodes a cytokine receptor that belongs to the interleukin 1 receptor family. This protein is a receptor for several cytokines involved in inflammatory responses. Therefore, the downregulation of *CD14* and *IL-1R* expression in *M. bovis*-infected animals suggests that the bacilli inhibit signaling pathways of antibacterial host defense. Moreover, the expression of *CD14* together with the thrombospondin 1 gene (*THSP1*) is repressed in CD4+ T lymphocytes cocultured with monocytes in response to *M. tuberculosis* as part of a suppression mechanism [18] induced by suppressor carbohydrates generated from CD8+ T cells.

Finally, the repressed *FYVE* gene encodes a domain found in various proteins including some implicated in vacuolar protein sorting and endosome function. The FYVE domain is implicated in signal transduction and membrane trafficking functions, such as stabilization of the interaction of early endosome antigen 1 with the small GTPase Rab5 [19]. However, FYVE domains might have additional functions [20].

Meade et al. [9] analyzed the expression profile of non-stimulated PBMCs from cattle infected with *M. bovis*. This study has revealed downregulation of the expression of key innate immune genes, including the toll-like receptor 2 (*TLR2*) and *TLR4*. Consistently with that finding, we detected reduced relative expression of *TLR2*, *TLR3* and *TLR4* in the *M. bovis*-infected group (−2.467-fold, −1.172-fold, −1.816-fold, respectively).

Two recent transcriptomic studies have deeply explored the global expression changes induced by *M. bovis* infection in a variety of blood cell populations [10], [21]. In one of these studies, Killick et al [10] identified the transcriptional profile of peripheral blood leukocytes from cattle infected with *M. bovis*, finding that the top ranking canonical pathway was involved in the inflammatory response. Importantly, several genes involved in TLR-mediated signaling and host immune-related genes that have displayed reduced relative expression in the study of Killick et al. were also found to be downregulated in the current study. For instance, *MYD88* (−0.765-fold), *MAPK13* (−1.780-fold), *MAPK14* (−0773-fold), *TREM1* (−4,011-fold), *TYROBP* (−2.602-fold), *DEFBIO* (−1.144-fold), *IL-16* (−1.245-fold) and *IL-18* (−1.304-fold).

In its recent study, Aranday-Cortes have found that the expression of *CXCL9*, *CXCL10*, *GZMA* and *IL-22* was significantly increased in PBMC from infected cattle compared to naïve animals following PPD stimulation [22]. In the current study we found that the expression of *CXCL10* was highly upregulated and the expression of *GZMA* was downregulated in infected animals, but the microarray data of these genes did not pass the statistical test. Probes sets for either *CXCL9* or *IL-22* were not represented in the microarrays used in this study; therefore we failed to assess the expression of these genes.

Previous studies on gene transcription profiling in peripheral blood cells of TB patients have identified the IFN signalling as the most significantly over-represented pathway [23], [24]. Although we found significant upregulation of the IFNγ, most of the downstream genes of IFN signaling pathways showed a reduced relative expression in infected animals. In fact, the expression of *IFNGR2*, the signal transduction chain of IFNγ receptor, was significantly downregulated in the infected group, suggesting a functional blockade of some aspects of IFNγ signaling. It has been reported that the IFNGR2 chain is constitutively expressed, but its expression level may be tightly regulated according to the state of cellular differentiation or activation [25]. For example, IFNγ can produce growth inhibition of some CD4+ Th1 cell populations [25]. Thus, downregulation of IFNGR2 would block growth-inhibitory effects of IFNγ, allowing the Th1 cell proliferation, which is consistent with a bTB immune-profile.

The MHC loading class II transactivator, *CIITA*, the transporter associated with antigen processing *TAP*, the major histocompatibility complex class II DM alpha-chain *HLA-DMA*, the *BLA-DQB* MHC class II antigen, and the bovine major histocompatibility complex class II genes (BOLA) are some of the identified upregulated genes in *M. bovis* infected cattle (Table S1). These results indicate that one of the cell effects triggered by IFNγ, the MHC I and II antigen presentation pathway, is clearly induced in infected animals in spite of the downregulation of the specific IFNγ signal transduction pathways.

In conclusion, the results presented here suggest that the differentially-expressed genes identified using microarray analysis and validated in additional samples by qRT-PCR play a role in disease pathogenesis. Therefore, these genes may serve as biomarkers for bTB-infection status. However, it is important to take into consideration that some of the gene expression changes observed in this study may not be specific for *M. bovis* infection and may represent a shared transcriptional program induced by infectious diseases. Thus, further studies are needed to investigate this possibility.

## Materials and Methods

### Ethics Statement

Animal experimentations were performed inside the biosafety facilities of the National Institute of Agricultural Technology (INTA), Argentina, in compliance with the regulations of Institutional Animal Care and Use Committee (IACUC) of INTA and authorized by the National Service of Agricultural and Food Health and Quality (SENASA) and National Consultant Commission of Agricultural Biotechnology (CONABIA). Ethical approval for the study was obtained from IACUC (n° 18/2011).

### Infection and Sampling

Samples for the microarrays and RT-qPCR experiments were taken from two independent *M. bovis* experimental infections of Fresian calves (nine-ten months old). Calves were negative for the tuberculin skin test and showed absence of an in vitro gamma interferon (IFNγ) response to both avian tuberculin PPD (PPDA) and bovine tuberculin PPD (PPDB), but all turned positive for tuberculin skin test and in vitro gamma interferon (IFNγ) response to PPDB at 45 days post-infection. Groups of animals were inoculated intratracheally with 10^7^ CFU of *M. bovis,* as described previously [26]. The disease status of the animals was examined at post-mortem (four months post-infection) by the presence of lesions typical of bTB in lungs, liver, and retropharyngeal, mediastinal, and tracheobronchial lymph nodes (data not shown). Blood samples for RNA extraction were taken a week before euthanasia. Although two of the animals sampled for microarray experiments did not show lesions, *M. bovis* culture was positive in lymph nodes and lungs in all animals (data not shown). All the animals sampled for RT-qPCR analysis showed many lesions in lungs and lymph nodes compatible with bTB (data not shown).

For control samples of microarray experiments, healthy cattle were selected from a herd without a recent history of bTB infection. Animals included in this control group were negative for IFNγ ELISA (IFNγ +, Bovigam) and tuberculin skin test (data not shown).

Heparinized blood (10 ml) from each animal was used for PBMC isolation by gradient centrifugation over histopaque 1077 (Sigma Aldrich) following the manufacturer’s protocol. PBMCs were incubated at 37°C in Roswell Park Memorial Institute (RPMI) complete medium supplemented with 10% of bovine fetal serum (Internegocios) and 20 µg/mL final concentration of PPDB (Biocor) on 12-well tissue culture plates for 16 h for RNA extraction.

### Microarray Hybridization

Eleven microarray slides, representing five biological replicates for PBMCs from PPD−/IFNγ - calves (healthy animals) and six for PBMCs from *M. bovis*-infected calves, were processed. The Affymetrix GeneChip® Bovine Genome Array platform (Affymetrix, Santa Clara, USA) was used in this study. The array contains 24,027 probe sets representing over 23,000 transcripts from *Bos taurus* and includes approximately 19,000 annotated UniGene clusters. The experiment was designed to be in compliance with Minimum Information About a Microarray Experiment (MIAME) standards. Each RNA sample was processed and hybridized to individual slides. Target preparation including verification of RNA quality assessed using a Bioanalyzer 2100 (Agilent Technologies, Santa Clara, USA) and microarray processing procedures were carried out at the Affymetrix facility of the School of Agronomy of the University of Buenos Aires, as described in the Affymetrix GeneChip Expression Analysis Manual (Affymetrix), and scanning was performed with a Microarray Scanner 3000 7G (Affymetrix).

### Statistical Analysis of Microarray Data

The analysis of expression data was performed with different packages from the Bioconductor project (http://www.bioconductor.org), an extension for bioinformatics of the R statistical language (http://r-project.org). The data quality of 11 CEL files obtained from the Affymetrix chips was assessed with the affyPLM [7], [27] and simpleaffy packages. The raw expression data were normalized using the function rma of the package Affy [28], which replicates the standardization procedure of the Affymetrix’s MicroArray Suite (MAS) software (the workflow of statistical analysis of microarray data is described in Table S2). Probe sets lacking an NCBI Entrez Gene identifier were removed. In the case of multiple probe sets mapping the same Entrez Gene ID, only the probe set with the largest variation across samples was retained. The remaining data were filtered by removing probe sets with a non-significant difference between treatments determined by a t-test with a p-value of 0.05 (not corrected for multiple comparisons). The Bioconductor limma package [29] was used for statistical analysis of the expression profiles and the GSEABase package [29] for gene set enrichment analysis.

### Validation of Microarray Results by RT-qPCR

The microarray data were validated by RT-qPCR using PBMC s collected from eight animals different from those sampled for the microarray experiments. Cells were obtained and processed as above. DNA-free RNA (1 µg) was mixed with 50 ng of random primers (Invitrogen) in 20 µl of final volume and reverse-transcribed to total cDNA with SuperScript II reverse transcriptase (Invitrogen) following the manufacturer’s instructions. cDNA (1 µl) was used as template for each real time quantitative PCR (RT-qPCR) reaction. All primers were designed using Primer Express Software to span an intron-exon boundary and to anneal only to cDNA synthesized from spliced mRNAs. Primer sequences are listed in Table S3. qPCR reactions were performed as described by Blanco et al. [30]. Briefly, SYBR green QuantiTec Mastermix (Qiagen) was used and reactions were made on Applied Biosystems 7000 SDS using standard cycling conditions. All reactions were performed in duplicate, and qPCR data were analyzed using the 2-ddCT with efficiency correction as described previously [31] to assess differences on gene expression in macrophages within groups. The *gadph* gene was used as control gene and data from the control group were used as the calibrator. REST beta 9 software (http://www.gene-quantification.info/, rest-mcs-beta-9august 2006) was used for final calculations and statistical analysis [31].

## Supporting Information

Figure S1 **Data cleaning and analysis workflow.** After normalization the expression data were cleaned using functions from the Bioconductor’s genefilter library. The probe sets that passed the filtering steps were analyzed with two different procedures. First, probe sets with a significant differential expression that was corrected for multiple tests were searched with the limma procedure. Secondly, the KEGG annotations of the probe sets were used to discover metabolic signaling or other pathways that showed differential expression.
(PPT)

Table S1 **Total differential expressed genes between PBMCs from infected animals and PBMCs from healthy animals.** FC: log2-fold change. F is the value of the statistics obtained with the limma procedure (linear models for microarray data; see Materials and Methods).
(XLSX)

Table S2 **Total differential pathways between PBMC from** ***M. bovis*** **-infected animals and control animals using GSEA.** The differential pathways were assessed with the gseattperm function in the Category Bioconductor package (10,000 permutations). All the pathways shown were significant at a P<0.01, adjusted for multiple comparisons using the false discovery rate method.
(XLSX)

Table S3 **Target Genes and Primer Sequences.** Primer pairs of selected genes shown in Figure1. *gadph* is the calibrator gene.
(DOCX)
