# Supplementary material for: Transcriptional Response of Peripheral Blood Mononuclear Cells from Cattle Infected with Mycobacterium bovis
Source: PLoS One. 2012 Jul 16;7(7):e41066. doi: 10.1371/journal.pone.0041066 (PMC3397951; doi:10.1371/journal.pone.0041066)
Supplement: Figure S1 — Data cleaning and analysis workflow. After normalization the expression data were cleaned using functions from the Bioconductor’s genefilter library. The probe sets that passed the filtering steps were analyzed with two different procedures. First, probe sets with a significant differential expression that was corrected for multiple tests were searched with the limma procedure. Secondly, the KEGG annotations of the probe sets were used to discover metabolic signaling or other pathways that showed differential expression. (PPT) [file pone.0041066.s001.ppt]

## Slide 1
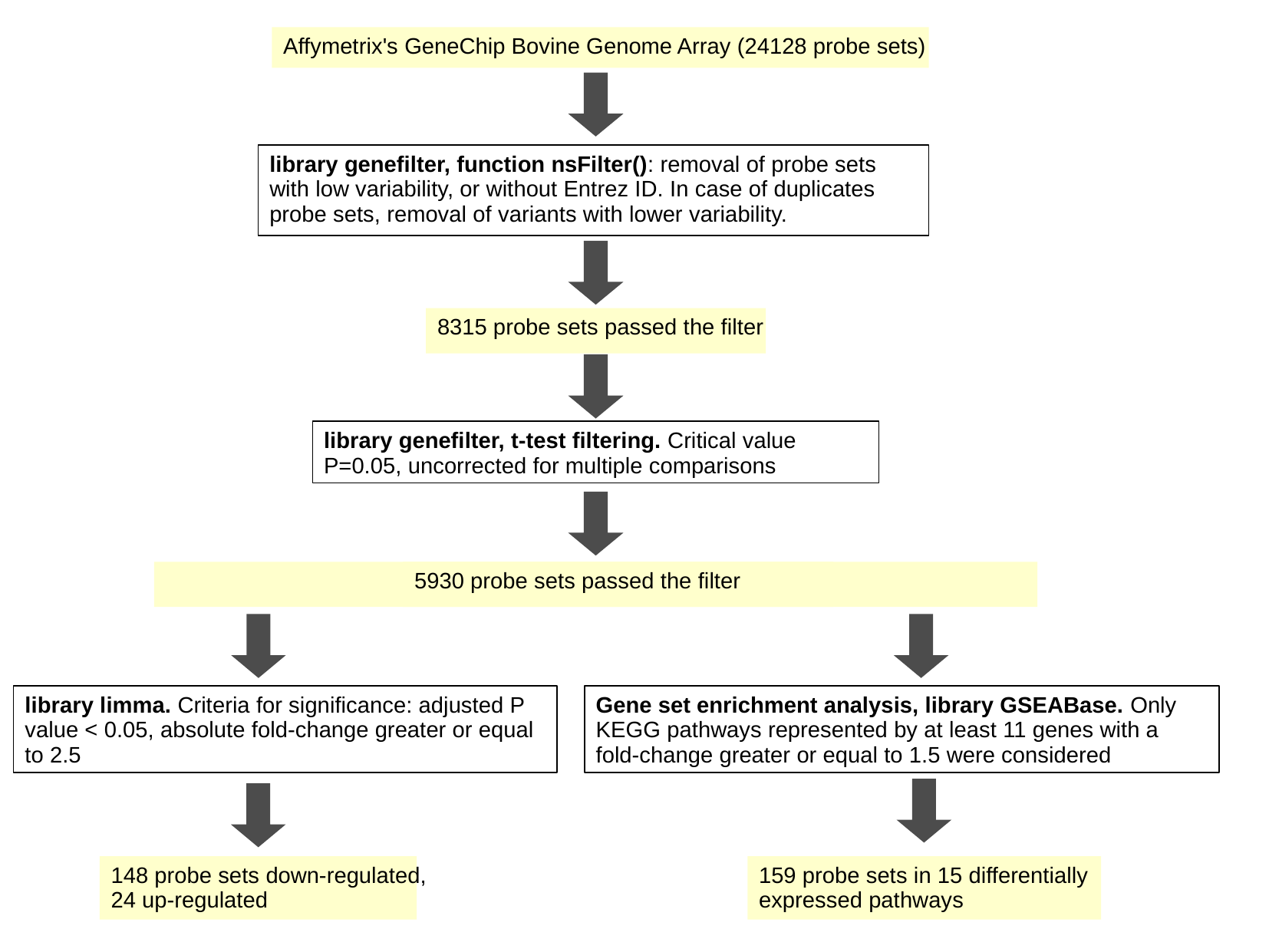

Affymetrix's GeneChip Bovine Genome Array (24128 probe sets)
library genefilter, function nsFilter(): removal of probe sets with low variability, or without Entrez ID. In case of duplicates probe sets, removal of variants with lower variability.
8315 probe sets passed the filter
library genefilter, t-test filtering. Critical value P=0.05, uncorrected for multiple comparisons
5930 probe sets passed the filter
library limma. Criteria for significance: adjusted P value < 0.05, absolute fold-change greater or equal to 2.5
Gene set enrichment analysis, library GSEABase. Only KEGG pathways represented by at least 11 genes with a fold-change greater or equal to 1.5 were considered
148 probe sets down-regulated,
24 up-regulated
159 probe sets in 15 differentially
expressed pathways
