# Supplementary material for: Transcriptional Response of Peripheral Blood Mononuclear Cells from Cattle Infected with Mycobacterium bovis
Source: PLoS One. 2012 Jul 16;7(7):e41066. doi: 10.1371/journal.pone.0041066 (PMC3397951; doi:10.1371/journal.pone.0041066)
Supplement: Table S3 — Target Genes and Primer Sequences. Primer pairs of selected genes shown in Figure1. gadph is the calibrator gene. (DOCX) [file pone.0041066.s004.docx]

**Primers used in this study**

| ***Primers*** | ***Sequences*** | ***Tm (ºC)*** |
| --- | --- | --- |
| CD14 | **F**: GCCCAGTGCACTAAGGTCTC  **R**: GCTCCCTGCTTAGCTTGTTG | 82.2 |
| FYVE | **F**: GGTCATCTGTGGGAAGTGCT  **R**: CCTCAAGGCTGAGGTTCAAG | 81.7 |
| IL-1R1 | **F**: TCACCAGCACAAAGACAAGC  **R**: TAGGCTCATGCTGCACAAAC | 77.7 |
| MMP9 | **F:** TAGCACGCACGACATCTTTC  **R:** GAAGGTCACGTAGCCCACAT | 80.0 |
| THBS1 | **F**: GGCACAAATAGCTCCACCAT  **R**: AGCTCCGGTGAGTTCAAAGA | 83.6 |
| NOLC1 | **F:** AAGTTGTGGCTAAGGCTCCA  **R:** GGGCTTTTTCTTCTGCTCCT | 80.7 |
| MHCII | **F**: ACAGTGACCATCTCCCCATC  **R**: AACCACCGAACCTTGATCTG | 80.8 |
| IL-2 | **F:** CGTGCCCAAGGTTAACGCTA  **R:** CCATTGAATCCTTGATCTCTCTGG | 76.0 |
| IFNγ | **F:** AGCTGATTCAAATTCCGGTGG  **R:** GATTTTGGCGACAGGTCATTC | 77.4 |
| GAPDH | **F:** ATCTCTGCACCTTCTGCCGA | 79.0 |
|  | **R:** GCAGGAGGCATTGCTGACA |  |
